# A dataset for assessing real-time attention levels of the students during online classes

**DOI:** 10.1016/j.dib.2023.109771

**Published:** 2023-11-04

**Authors:** Muhammad Kamal Hossen, Mohammad Shorif Uddin

**Affiliations:** a Department of Computer Science and Engineering, Jahangirnagar University, Dhaka 1342, Bangladesh; b Department of Computer Science and Engineering, Chittagong University of Engineering and Technology, Chattogram 4349, Bangladesh

**Keywords:** Face detection, Hand tracking, Head pose, MediaPipe, Mobile phone, Machine learning, Classification

## Abstract

This dataset offers a comprehensive compilation of attention-related features captured during online classes. The dataset is generated through the integration of key components including face detection, hand tracking, head pose estimation, and mobile phone detection modules. The data collection process involves leveraging a web interface created using the Django web framework. Video frames of participating students are collected following institutional guidelines and informed consent through their webcams, subsequently decomposed into frames at a rate of 20 FPS, and transformed from BGR to RGB color model. The aforesaid modules subsequently process these video frames to extract raw data. The dataset consists of 16 features and one label column, encompassing numerical, categorical, and floating-point values. Inherent to its potential, the dataset enables researchers and practitioners to explore and examine attention-related patterns and characteristics exhibited by students during online classes. The composition and design of the dataset offer a unique opportunity to delve into the correlations and interactions among face presence, hand movements, head orientations, and phone interactions. Researchers can leverage this dataset to investigate and develop machine learning models aimed at automatic attention detection, thereby contributing to enhancing remote learning experiences and educational outcomes. The dataset in question also constitutes a highly valuable resource for the scientific community, enabling a thorough exploration of the multifaceted aspects pertaining to student attention levels during online classes. Its rich and diverse feature set, coupled with the underlying data collection methodology, provides ample opportunities for reuse and exploration across multiple domains including education, psychology, and computer vision research.

Specifications Table

**Table:** 

Subject	Computer Science
Specific subject area	Students Attention Monitoring, Online Learning, Computer Vision, and Machine Learning
Data format	Raw: csv file
Type of data	Table
Data collection	The data collection process involved integrating face detection, hand tracking, head pose estimation, and mobile phone detection modules into an online class environment. Video frames, obtained with informed consent from students, were captured from webcams at 20 FPS through a custom Django-based web interface between 1st and 30th June 2023. Subsequently, these frames were processed by the specified modules to extract raw data. Face detection was implemented using the MediaPipe Face Detection solution based on BlazeFace. Hand tracking utilized the MediaPipe Hands solution, while head pose estimation relied on the MediaPipe Face Mesh solution and the solvePNP() of OpenCV. Mobile phone detection was accomplished through the YOLOv7 model with PyTorch. Data normalization techniques such as one-hot encoding and maximum absolute scaling were applied to specific variables during preprocessing.
Data source location	Institution: Jahangirnagar University City: Dhaka Country: Bangladesh Latitude and longitude (and GPS coordinates, if possible) for collected samples/data: 23°52′55.6″N and 90°16′02.5″E
Data accessibility	Repository name: Mendeley Data Data identification number (DOI):10.17632/smzggbnkd2.1 Direct URL to data: https://data.mendeley.com/datasets/smzggbnkd2/1

## 1 Value of the Data

• The significance of this dataset lies in its exclusive focus on a critical facet of education namely the attention levels of students during online classes. By capturing real-time attention dynamics, it provides a unique opportunity to explore a domain directly impacting learning outcomes.
• The dataset presented here is a valuable asset for researchers, educators, and psychologists interested in understanding the nuances of attention patterns in the realm of online education. The data can inform the development of effective teaching strategies tailored to individual students and the creation of supportive learning environments. By comprehensively analyzing attention-related features in online classes, this resource provides the foundation for data-informed educational improvements. It empowers educators and institutions to design personalized interventions, ultimately enhancing the overall quality of virtual teaching and learning experiences while promoting active engagement among students.
• Beyond its primary purpose, this dataset serves as a benchmark for refining attention detection algorithms. Researchers can leverage this resource to rigorously assess and enhance diverse algorithms. Through robust cross-validation techniques, these algorithms can be fine-tuned to enhance precision and effectiveness in quantifying attention levels. This iterative process empowers the research community to develop and validate innovative methods for automated attention detection, resulting in improved educational technology. The dataset not only supports advancements in the quantification of attention but also fosters innovation in the broader field of machine learning, benefiting various domains requiring nuanced data analysis.
• This dataset offers diverse research prospects, allowing scholars to delve into complex associations. It enables academics to investigate the connections between attention patterns and educational outcomes. By meticulously examining the impact of instructional strategies, environmental factors, and technological distractions on student engagement and comprehension, this resource provides valuable insights into the multifaceted aspects of online learning.
• The accumulated data paves the way for practical applications, particularly the development of real-time attention monitoring tools. These tools have the potential to empower educators by providing them with timely insights into student attention. In turn, educators can adapt their teaching techniques based on real-time attention indicators, fostering interactive and responsive learning environments that promote active participation and heightened engagement.
• By providing a meticulously curated and standardized collection of data, this resource opens the door to catalyze innovation within the expansive realm of educational technology. Scholars and researchers alike are presented with a unique opportunity to delve into novel characteristics and cutting-edge algorithms associated with attention detection. This, in turn, serves as a driving force for advancing the entire field of automated attention detection, leading to the development of more sophisticated and accurate models. Moreover, this innovative potential has a ripple effect, resulting in a significant enhancement of the overall efficiency and efficacy of online educational platforms.

## 2 Background

The primary aim of this study is to present a comprehensive and multifaceted dataset assessing student attention levels in online classes, featuring face detection, hand tracking, phone usage, and head pose estimation. Another important objective of this paper is to offer a valuable resource for studying the impact of attention patterns on virtual learning outcomes, advancing online teaching methods, and fostering educational technology innovation by providing benchmark data for attention-detection algorithms and real-time monitoring tools to enhance online education quality.

## 3 Data Description

In an era dominated by digital learning, online classes have become a ubiquitous mode of education and ensuring students remain engaged and attentive during these virtual sessions presents a substantial challenge for educators. However, limited datasets capturing attention dynamics during online classes hinder research and innovation to address this challenge. A dedicated dataset addressing this gap is essential to unravel the nuances of attention patterns, enabling educators and researchers to optimize teaching methods, devise interventions, and develop technology-driven solutions. This dataset bridges the gap between attention and learning, facilitating a deeper comprehension of engagement and interactions of the students in the online classroom environment.

As stated above, the developed dataset [1] is designed to facilitate the analysis of attention and engagement behaviors of students through various features extracted from multi-modal data sources. The dataset consists of 17 columns, with 16 columns dedicated to features derived from different detection and estimation modules, and one column serving as the label indicating attention status. Each row represents a session, capturing interactions and behaviors of students during educational activities. Below is a concise description of the feature and label columns of the dataset with data type in Table 1.

**Table 1:** Summary of features and label with data type.

No.	Feature	Description	Data Type
1	no_of_face	Number of faces found in the frame	Integer
2	face_x	X-coordinate of the upper-left corner of the face	Float
3	face_y	Y-coordinate of the upper-left corner of the face	Float
4	face_w	Width of the face (in pixels)	Float
5	face_h	Height of the face (in pixels)	Float
6	face_con	Face detection confidence	Float
7	no_of_hands	Number of detected hands	Integer
8	pose	Face orientation (Forward | Down | Left | Right)	Categorical
9	pose_x	Face orientation in x-axis	Float
10	pose_y	Face orientation in y-axis	Float
11	phone	Presence of a mobile phone (0 for absent, 1 for present)	Integer
12	phone_x	X-coordinate of the upper-left corner of the detected phone	Float
13	phone_y	Y-coordinate of the upper-left corner of the detected phone	Float
14	phone_w	Width of the phone (in pixels)	Float
15	phone_h	Height of the phone (in pixels)	Float
16	phone_con	Phone detection confidence	Float
17	label	Target Column (0: Attentive, 1: Inattentive)	Integer

Table 2 offers a succinct summary of attention labels in the dataset, presenting a binary classification of attention states that provides valuable insights into participation and engagement of students during the learning process.

**Table 2:** Brief details of attention labels.

Class Name	Description	Visualization	Features Value
Attentive	The 'Attentive' class refers to instances where student's exhibit focused and attentive behavior during online classes. These instances are characterized by the factors such as consistent face presence, appropriate head pose alignment, and minimal phone usage. This class signifies active participation and engagement with the learning material, contributing to a positive and productive learning environment.		[1, 230.34, 296.95, 156.42, 156.42, 94.94, 0, 'forward', −0.37, 7.39, 0, 0, 0, 0, 0, 0]
Inattentive	The 'Inattentive' class refers to instances where students display a lack of engagement during online classes. These instances typically involve factors such as turned-away faces, frequent head movement indicating distractions, reduced or absent hand tracking, and potential phone interactions. These indicators collectively suggest diminished attention and participation, highlighting potential distractions or disinterest in the learning content.		[1, 245.03, 275.44, 120.44, 120.44, 87.69, 0, 'down', −15, 10, 1, 208, 314, 315, 428, 0.86]

A comprehensive tabular representation is provided in Table 3 that summarizes essential statistics of the dataset, including measures like mean, standard deviation, minimum, median, and maximum, for some numerical columns. This offers an insight into the distribution of various important features.

**Table 3:** Summary statistics of numerical features.

Feature	Mean	Std. Dev.	Min.	Median	Max.
face_x	259.25	39.55	38.43	259.94	436.37
face_y	260.17	48.96	−29.02	271.65	434.43
face_w	162.30	38.76	88.25	153.91	486.32
face_h	162.30	38.75	88.25	153.91	486.34
face_con	88.37	8.46	50.34	90.65	98.24
pose_x	−0.07	7.67	−19.98	1.11	32.58
pose_y	2.15	8.84	−38.62	3.61	36.57
phone_x	188.67	93.53	1.00	200.00	500.00
phone_y	293.36	92.14	78.00	306.00	447.00
phone_w	313.14	98.49	57.00	315.00	640.00
phone_h	446.17	55.29	107.00	478.00	480.00
phone_con	0.78	0.08	0.600	0.80	0.93

**Fig. 1:**
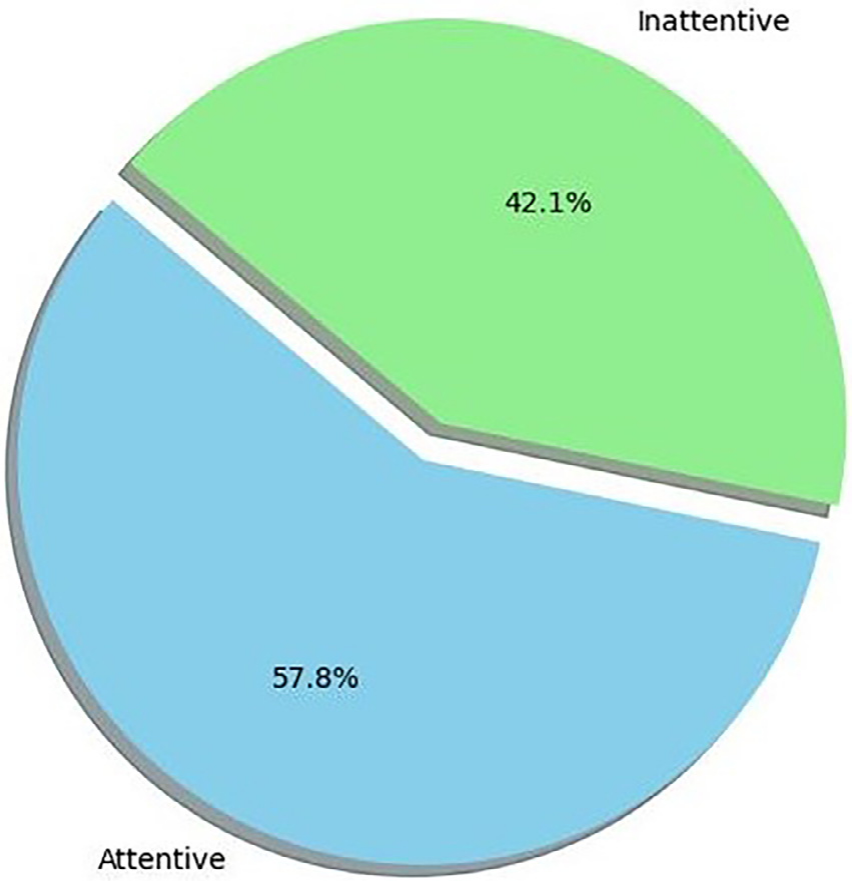
Distribution of attention labels.

The pie chart of Fig. 1 provides an overview of the balance of dataset and allows us to understand the proportion of attentive and inattentive instances in the dataset.

The dataset includes instances with varying counts of recognized faces and hands during online class sessions. Specifically, there are 28 instances with two detected faces, 3901 instances with one detected face, and 71 instances with no detected faces. In terms of detected hands, there are two hands detected in 975 instances, one hand detected in 763 instances, and no hands detected in 2262 instances.

**Fig. 2:**
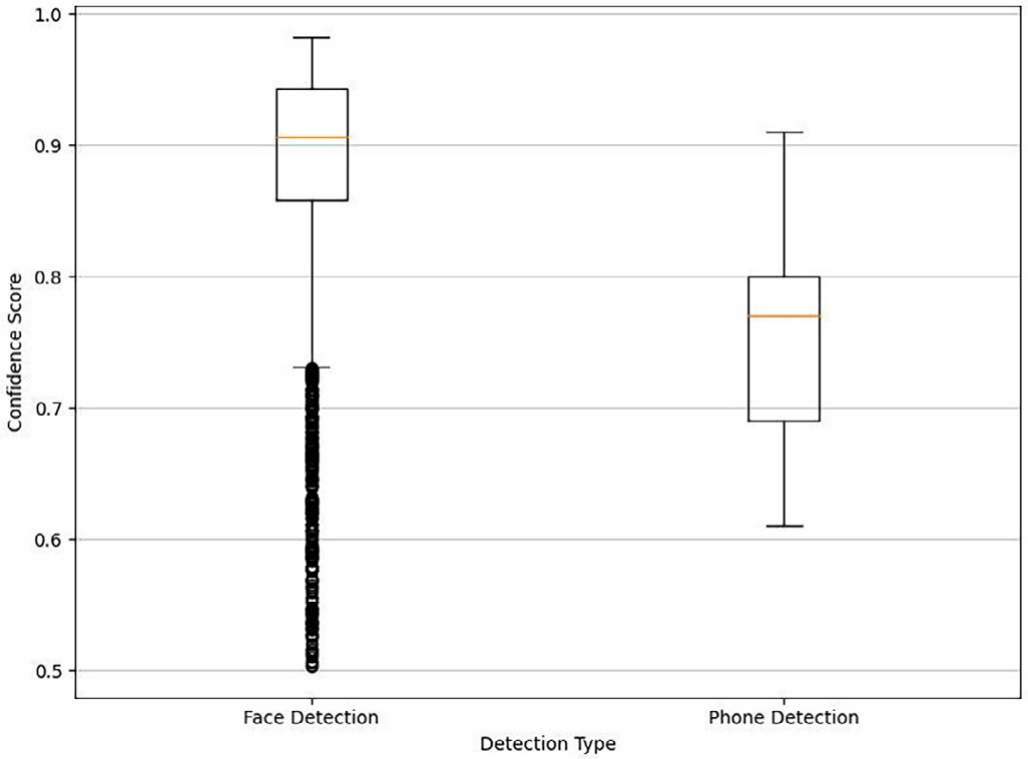
Confidence scores distribution for face and phone detection.

A box plot in Fig. 2 visualizes the distribution of confidence scores for the face detection and phone detection modules, providing insights into the quality of detections; it's noteworthy that many outlying scores for face detection are plotted individually.

**Fig. 3:**
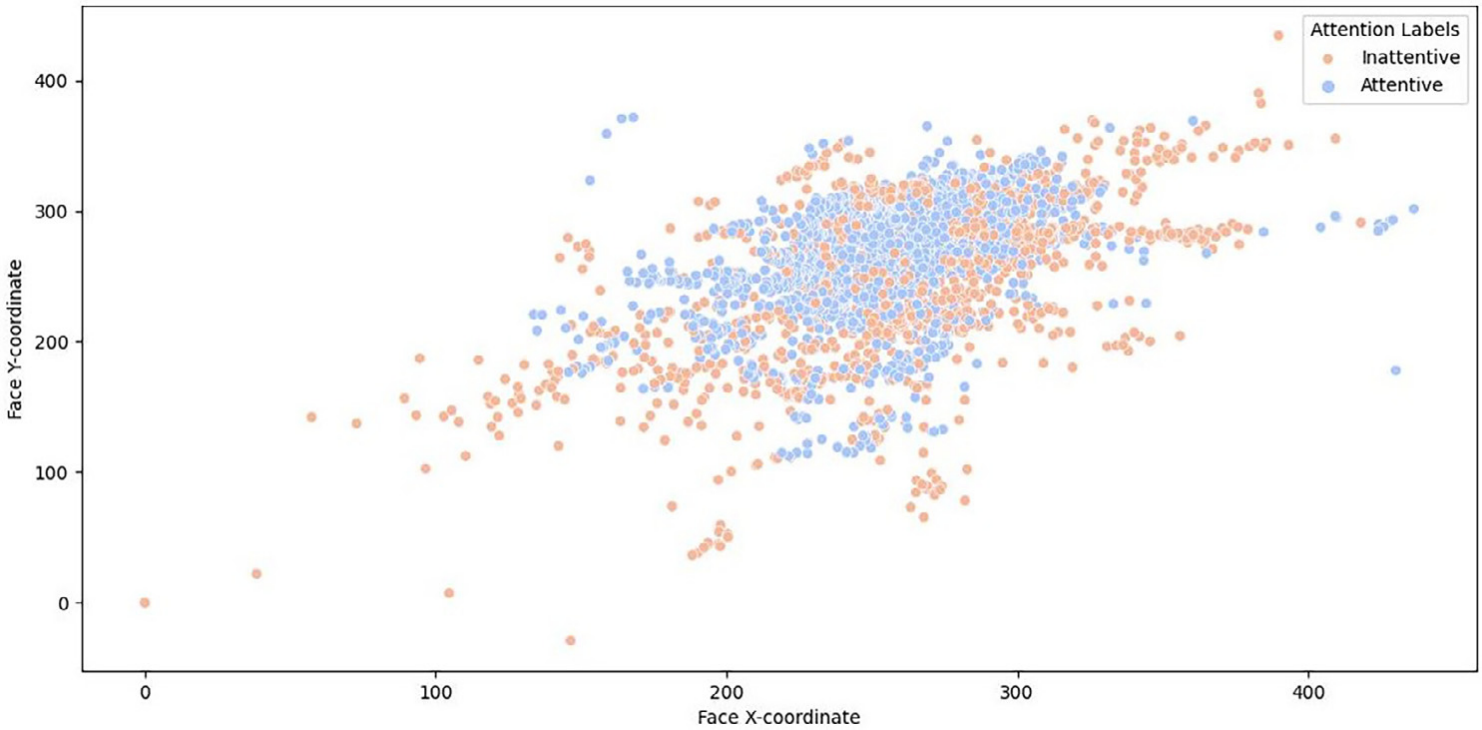
Relationship between detected face coordinates and attention labels.

Fig. 3 illustrates the correlation between detected face coordinates (face_x, face_y) and attention labels, uncovering potential patterns associated with face position and attention levels in the dataset.

Within the dataset, the ‘pose’ feature categorizes head orientation as ‘forward,’ ‘down,’ ‘left,’ and ‘right’ based on specific criteria related to ‘pose_x’ and ‘pose_y’ features in degrees, offering valuable insights into the association of students' head positioning during online classes with their attention levels. Specifically, the dataset exhibits 2951 occurrences of ‘forward,’ 537 of ‘down,’ 334 of ‘left,’ and 178 of ‘right’ categories.

The dataset is conveniently stored within the data repository as a consolidated file named *'attention_detection_dataset_v1.csv*'. As previously highlighted, this file encompasses a collection of 16 distinct features, encompassing both integer and float data types, alongside categorical values. Notably, the dataset incorporates a dedicated label column that plays a pivotal role in the classification process.

## 4 Experimental Design, Materials and Methods

### 4.1 Data acquisition

**Fig. 4:**
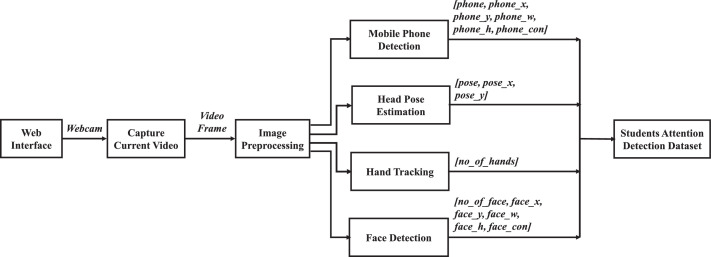
Overview of the dataset generation process.

A comprehensive overview of the dataset generation process is provided in Fig. 4, showcasing the integration of four fundamental components such as face detection, hand tracking, head pose estimation, and mobile phone detection module. Our approach involved the development of a user-friendly web interface built on the Django framework [2], ensuring a seamless and efficient data collection process. This interface facilitated the collection of video frames from students actively participating in online classes of a course of computer science. Maintaining uniformity, these video frames were extracted at a consistent and reliable rate of 20 frames per second (FPS) and meticulously transformed from the BGR to RGB color model. As part of our commitment to ethical data collection, every step of the process was conducted with strict adherence to pertinent legal regulations and institutional protocols. Prior to data collection, comprehensive informed consent was diligently sought from all participating students, ensuring their rights and privacy were upheld throughout the study. The subsequent sub-sections offer succinct insights into the role of each component and their collaborative contribution to capturing attention-related attributes of the students.

### 4.2 Face detection module

We designed a web interface to capture video frames of students engaged in online classes via their webcams. Subsequently, each captured frame undergoes meticulous analysis using our face detection module, which leverages the capabilities of the MediaPipe Face Detection solution [3], which is based on BlazeFace, to precisely localize faces and their corresponding facial attributes within the frame. The outcome of the face detector manifests as bounding boxes encompassing the identified facial regions within the image frame.

**Fig. 5:**
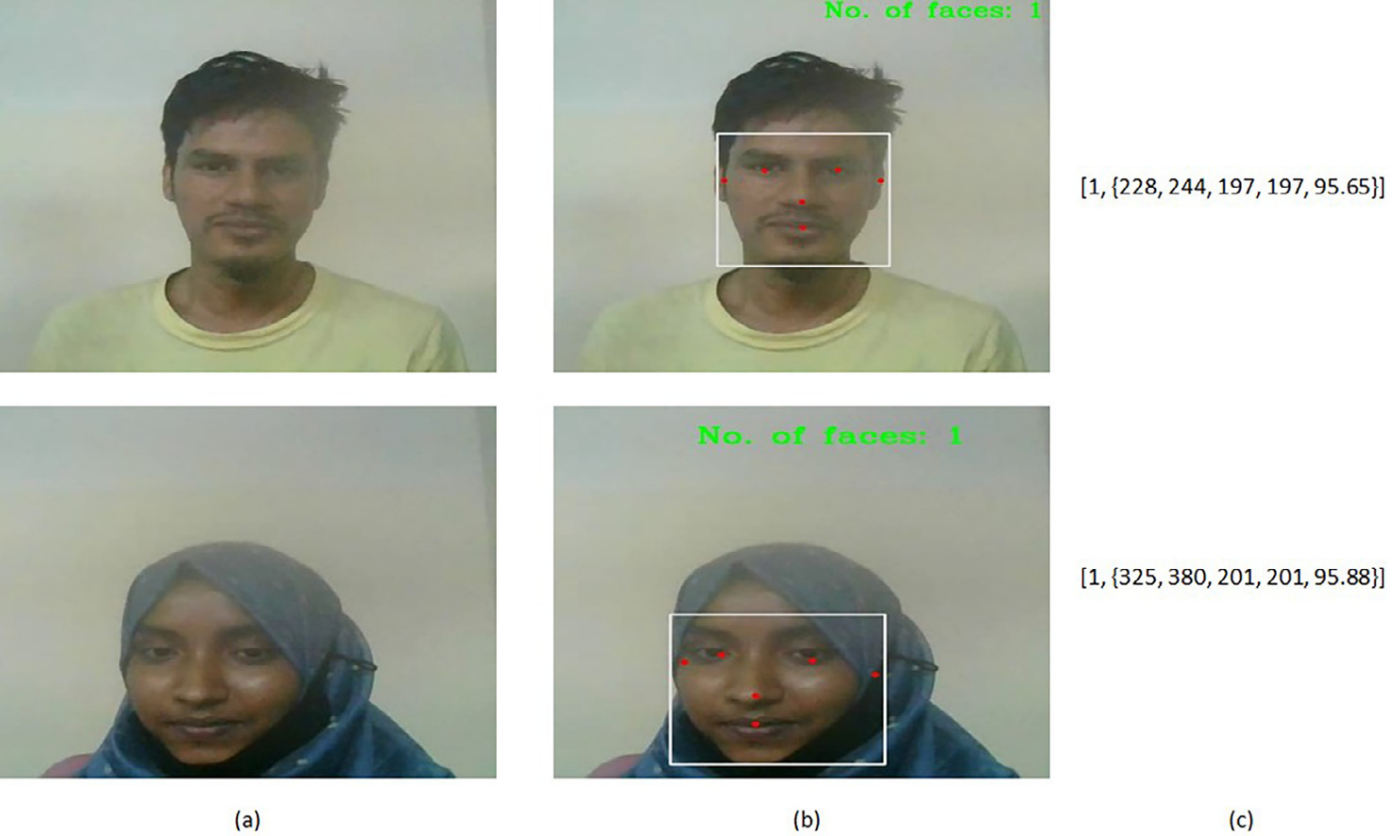
Face features collection: (a) Input frame, (b) Face detected image, and (c) Output features [no_of_face, {face_x, face_y, face_w, face_h, face_con}].

The face detection module computes the ‘no_of_face’ feature value within the frame by tallying the number of bounding boxes generated. Additionally, crucial facial characteristics of the detected face such as ‘face_x’, ‘face_y’, ‘face_w’, and ‘face_h’ feature values are extracted from attributes including ‘xmin’, ‘ymin’, ‘width’, and ‘height’ associated with the respective bounding box. To further enrich our feature set, the 'face_con' feature value can be derived from the 'score' attribute returned by the face detector, indicating the confidence level of the presence of the detected face. These pivotal feature values collectively contribute to a comprehensive understanding of the detected faces within the frame, as visually depicted in Fig. 5.

### 4.3 Hand tracking module

Following the processing conducted by the face detection module, the subsequent step involves subjecting each gathered frame to analysis via our hand-tracking module. This module makes use of the MediaPipe Hands solution [4], a sophisticated tool enabling the precise localization of key points pertaining to the hands. Furthermore, it facilitates the application of visual enhancements directly onto the hands within the frame, enhancing their visibility.

The outcome of the hand landmark model encompasses three essential components: firstly, the derivation of landmark coordinates of the detected hands in image space; secondly, the computation of landmark coordinates of the detected hands in a global reference frame (world coordinates); and lastly, the determination of hand dominance (handedness) for each detected hand. However, in our specific application, we exclusively focus on the landmark coordinates of the detected hands in image coordinates.

**Fig. 6:**
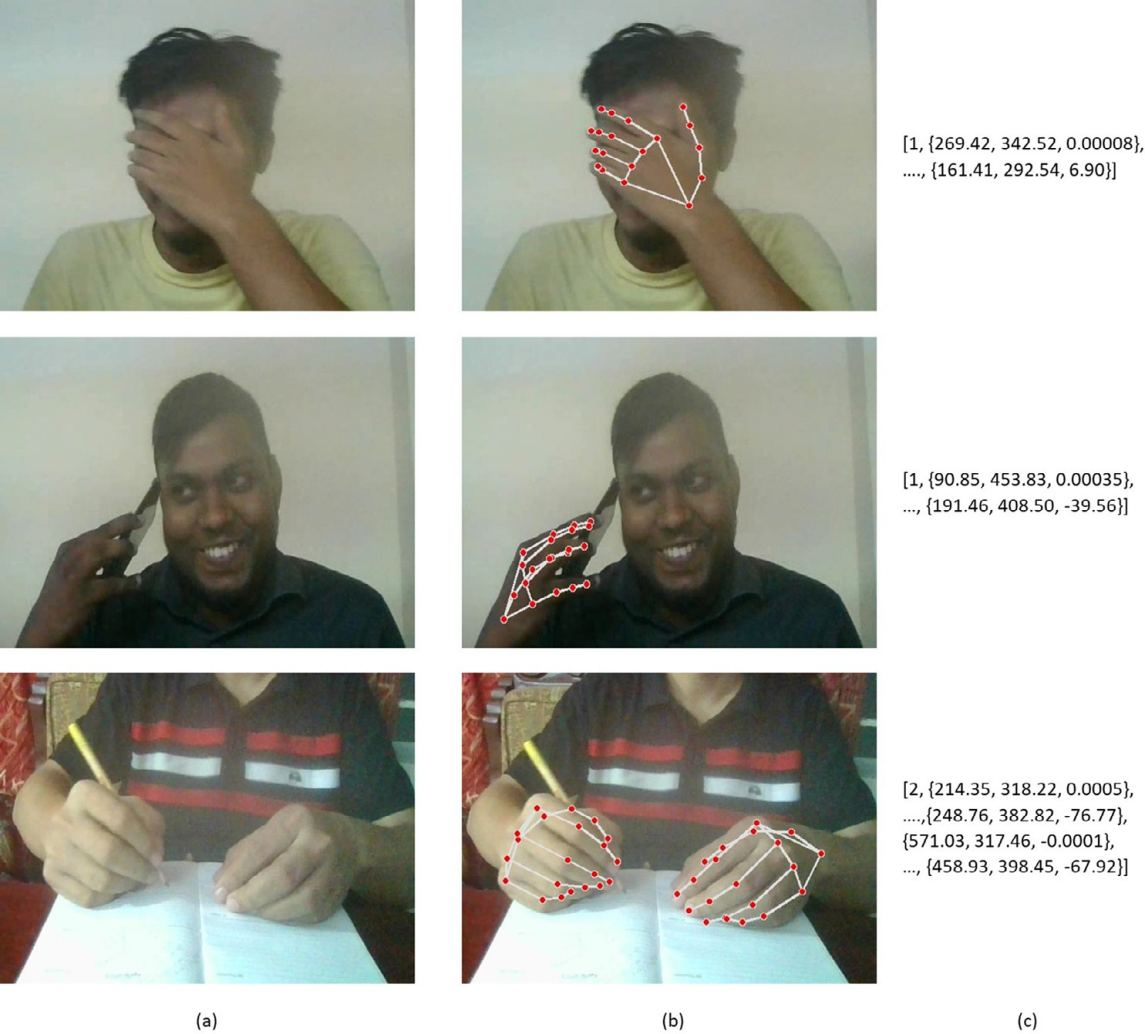
Hand tracking output: (a) Input frame, (b) Hands detected image, and (c) Output features [no_of_hands, hand landmarks {x, y, z}].

By employing these landmarks, we are able to accurately ascertain the number of hands that have been detected within the frame. This fundamental quantification of hands detected is then utilized to populate the 'no_of_hands' feature value with the corresponding count. A visual representation of this process can be found in Fig. 6, providing a clear illustration of the functioning of the hand tracking module and its contribution to the overall feature collection process. The figure displays the 3D coordinates of WRIST and PINKY_TIP landmarks, two of the 21 3D landmarks detected on the hand.

### 4.4 Head pose estimation module

Subsequent to this, each frame undergoes analysis within our head pose estimation module. This module leverages the MediaPipe Face Mesh [3] to discern all facial landmarks and employs the solvePnP() function from OpenCV [5] to deduce the pose of head. From the facial landmarks, we extract both 2-dimensional and 3-dimensional coordinates for key points such as the nose tip, chin, eye corners, and mouth corners. Utilizing properties of the webcam-obtained image, like focal length, center, and distortion coefficients, we compute the camera matrix. The rotation vector is then computed by applying the solvePnP() function, which takes in the 2D and 3D coordinates along with the camera matrix. This vector is transformed into a rotation matrix using the Rodrigues() function of OpenCV. Subsequently, the Euler angles of rotation are derived from this matrix through the RQDecomp3 × 3() function in OpenCV. These Euler angles encapsulate the pose of the head.

Thus, the head pose estimation module contributes to the dataset with the ‘pose’, ‘pose_x’, and ‘pose_y’ feature values, with ‘pose_x’ and ‘pose_y’ represented in degrees. Notably, the ‘pose’ feature takes on categorical values such as forward, down, left, and right, serving to classify the orientation of head according to specific criteria as stated below:

• If ‘pose_y’ is greater than 15°, the pose is labeled as ‘right.’
• If ‘pose_y’ is less than −10°, the pose is labeled as ‘left.’
• If ‘pose_x’ is less than −10°, the pose is labeled as ‘down.’
• Otherwise, the pose is labeled as ‘forward.’

**Fig. 7:**
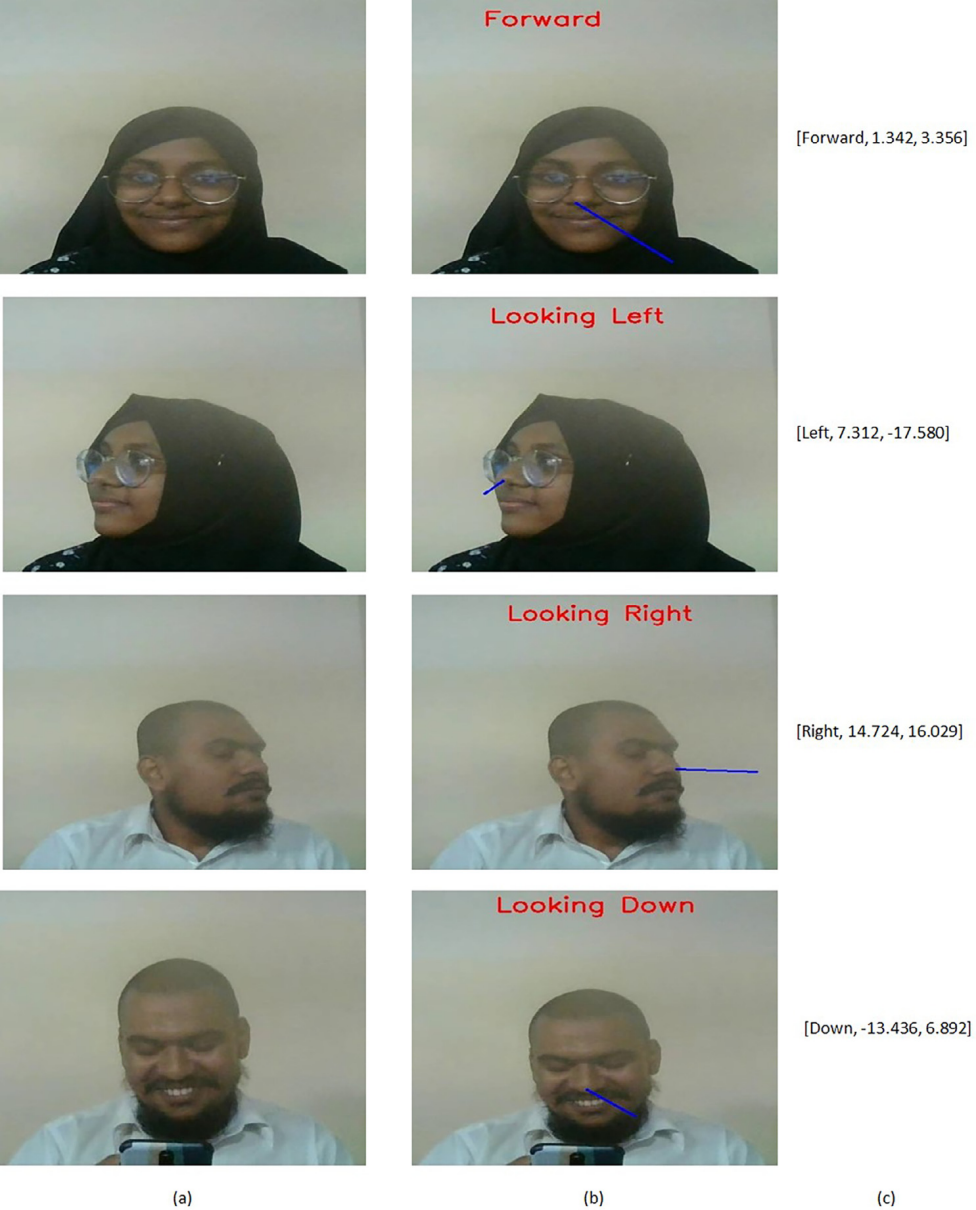
Head pose estimation results: (a) Input frame, (b) Estimated pose image, and (c) Output features [pose, pose_x, pose_y].

These stages are visually expounded in Fig. 7, encapsulating the essence of the head pose estimation process.

### 4.5 Mobile phone detection module

Following its processing by the head pose estimation module, each frame undergoes a conclusive analysis within our mobile phone detection module. This module capitalizes on the YOLOv7 [6], a real-time object identification algorithm of YOLO (You Only Look Once) family, to discern the presence of a mobile phone or cell phone within the frame. The YOLOv7, having been pretrained on the extensive MS COCO dataset [7] comprising over 200,000 labeled images and encompassing 80 distinct classes, including the “cell phone” class, is our chosen algorithm.

**Fig. 8:**
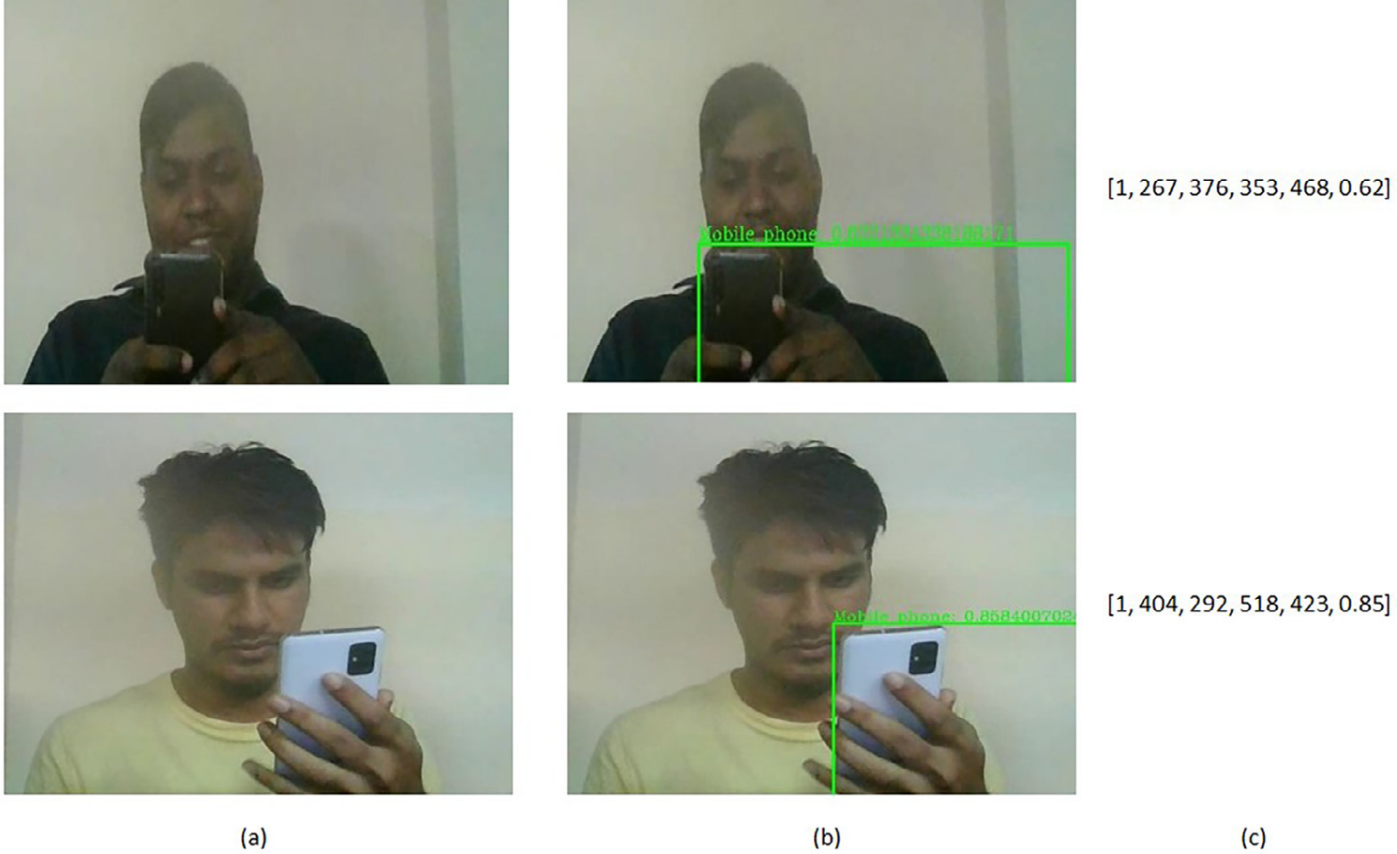
Outcome of mobile phone detection module: (a) Input frame, (b) Detected mobile phone image with confidence score, and (c) Output features [phone, phone_x, phone_y, phone_w, phone_h, phone_con].

In this process, the frame serves as input to the YOLOv7 model, which in turn delineates a bounding box around the identified mobile phone, subsequently furnishing high-level attributes such as x-coordinate, y-coordinate, width, height of the phone, and the confidence score of detection of the phone. To ensure precision, we apply a confidence score threshold of greater than 0.6, warranting the selection of an object as a mobile phone. Upon meeting this threshold, the ‘phone’ feature value is attributed a value of 1, thereby signifying the presence of a mobile phone. Consequently, the ‘phone_x’, ‘phone_y’, ‘phone_w’, ‘phone_h’, and ‘phone_con’ values within the dataset align with the corresponding attributes returned from the YOLOv7 model. The operational snapshot of this module within the visual context of the students is vividly portrayed in Fig. 8, encapsulating the essence of mobile phone detection within the overall framework.

### 4.6 Class labeling

Finally, we consider the following criteria as stated in Table 4 for assigning ‘0’ (Attentive) and '1' (Inattentive) labels to the attention detection dataset based on the information provided by the face detection, hand tracking, head pose estimation, and phone detection modules.

**Table 4:** Criteria for labeling the attention detection dataset.

Module	Criteria	Label
Face detection	When a face is confidently detected and centered within the frame.	0
	When no face is detected or the face is partially out of frame.	1
Hand tracking	When one or both hands are confidently detected and are within a reasonable distance from the face.	0
	When no hands are detected or the hands are too far from the face.	1
Head pose estimation	When the head pose is estimated as looking forward or slightly tilted and aligned with the screen.	0
	When the head pose is estimated as looking left, right, down or away from the screen.	1
Phone detection	When no phone is detected or the phone is placed face down on the table.	0
	When a phone is detected and positioned in a way that suggests distraction (e.g., screen facing up).	1

We also use a combination of different factors such as head pose, phone detection, hand count, and more to assign labels. For example, if both phone detection and head pose indicate inattention, we label the instance as Inattentive (1).

### 4.7 Data preprocessing

The dataset is designed for the evaluation and training of various machine learning algorithms, including Support Vector Machine (SVM), Decision Tree (DT), Random Forest (RF), AdaBoost, Gradient Boosting (GB), and Extreme Gradient Boosting (XGBoost) [8]. These algorithms aim to accomplish the task of attention detection, ensuring active engagement, participation, and overall improvement of students during online classes.

Categorical variables have been transformed into numerical equivalents using techniques like one-hot encoding or label encoding, making them compatible with machine learning algorithms. Some features have undergone maximum absolute scaling with MaxAbsScaler() function of Scikit-learn [9], individually adjusting each feature to a maximum absolute value of 1.0 within the dataset. Additionally, the 'face_con' values have been normalized to the [0.0, 1.0] range to facilitate processing. The dataset has been partitioned into training and testing subsets, maintaining an 80:20 split ratio, for effective model assessment.

## 5 Limitations

The effectiveness and applicability of the student's attention detection dataset could be constrained by certain limitations. Factors like lighting conditions, room arrangement, and device quality during data collection might introduce inconsistencies or biases in detections. While the size of the dataset is expected to increase in future versions, its current scope may be restricted, potentially affecting its capacity to encompass various scenarios and perform well on unseen instances. Furthermore, errors or inaccuracies in annotation or labeling might also compromise the reliability of the dataset. The slight imbalance between the instances of attentive and inattentive labels could lead to bias towards the more prevalent class. These constraints warrant careful consideration when utilizing the dataset for attention detection purposes.

## Ethics Statement

The data collection procedures adhered to all relevant laws and institutional guidelines. Explicit informed consent was obtained from all human participants, who willingly consented to the use of their images, including their face images, for purposes such as the publication of processed data, as described in this article. To protect privacy and confidentiality, all individuals in this dataset have been anonymized. The dataset is presented in its raw format, with thorough anonymization measures in place to address ethical and privacy considerations.

## CRediT authorship contribution statement

**Muhammad Kamal Hossen:** Conceptualization, Methodology, Data curation, Software, Writing – original draft, Visualization. **Mohammad Shorif Uddin:** Supervision, Validation, Writing – review & editing.

## Data Availability

Students Attention Detection Dataset (Original data) (Mendeley Data)
